# Moving in groups: how density and unpredictable motion affect predation risk

**DOI:** 10.1007/s00265-015-1885-1

**Published:** 2015-02-24

**Authors:** Nicholas E. Scott-Samuel, Gavin Holmes, Roland Baddeley, Innes C. Cuthill

**Affiliations:** 1 School of Experimental Psychology, University of Bristol, Bristol, UK; 2 School of Psychology, Cardiff University, Cardiff, UK; 3 School of Biological Sciences, University of Bristol, Bristol, UK

**Keywords:** Confusion effect, Predation risk, Aggregation, Group living, Object tracking, Visual search

## Abstract

One of the most widely applicable benefits of aggregation is a *per capita* reduction in predation risk. Many factors can contribute to this but, for moving groups, an increased difficulty in tracking and targeting one individual amongst many has received particular attention. This “confusion effect” has been proposed to result from a bottleneck in information processing, a hypothesis supported by both modelling and experiment. If the competition for limited attention is localised to the particular part of the visual field where the target is located, prey density is likely to be the key factor rather than group numbers *per se*. Furthermore, unpredictability of prey movement may enhance confusion, but both factors have received insufficient attention from empiricists: undoubtedly because of the difficulty of experimental manipulation in natural systems. We used a computer-based target tracking task with human subjects to manipulate effects of number and density independently, in factorial combination with motion path predictability. Density, rather than number, drove the confusion effect in our experiment and acted synergistically with the unpredictability of the direction of motion. The experimental paradigm we present offers the potential for isolating other factors affecting predation success on group-living prey, and forging links with the psychological literature on object tracking and visual search.

## Introduction

Whilst an increase in numbers can make a group more conspicuous, and therefore more easily detectable (e.g. Jackson et al. 2005; Ioannou and Krause 2008), there is evidence that moving as a group has advantages. One such advantage is the confusion effect. This is a hypothesised “reduced attack-to-kill ratio experienced by a predator resulting from an inability to single out and attack individual prey in a group” (Krause and Ruxton 2002). The predator confusion is generally attributed to a cognitive bottleneck acting at multiple levels of processing (Krakauer 1995). The confusion effect scales with the size of a target group (e.g. Treherne and Foster 1982; Krause and Ruxton 2002; although see Fels et al. 1995). Here, we concentrate on two factors which could enhance the confusion effect: predictability of the motion paths of individuals within a group, and overall group density.

Evidence about the relative effects of number and density is equivocal. Ioannou et al. (2008) found no density effect on attack rate in a stickleback-*Daphnia* system (*Gasterosteus aculeatus*, *Daphnia magna*), although they only varied density across two levels for one area; in contrast, Ioannou et al. (2009) reported that higher local density within a group increased attack error in the same stickleback-*Daphnia* system. In terms of prey preference (rather than predator behaviour), Frommen et al. (2009) showed that density can influence the preference of sticklebacks (*G. aculeatus*) when selecting a shoal to join. Furthermore, it has been demonstrated that both cyprinid fish (Krause 1993) and herring (*Clupea harengus*) (Domenici et al. 2000) shoals compact under threat.

Parallel research, using human predators and a computer-based capture task, concluded that the degree of unpredictability of the paths of objects in a group reduces capture performance, but does not interact with the confusion effect (Jones et al. 2011). This is somewhat unexpected, and given that unpredictable motion is an intuitively obvious and much discussed escape tactic (Driver and Humphries 1988; Ruxton et al. 2004) it is surprising that there have not been more experiments on the effectiveness of such behaviour in group-living prey.

This concept of a confusion effect resonates with a large literature on visual search tasks in humans (Wolfe 2010; Eckstein 2011), as noted by Tosh et al. (2009). This fact, and the promise of tighter experimental control over variables of potential interest, has led to the use of human subjects in experiments investigating the confusion effect (e.g. Ruxton et al. 2007; Jones et al. 2011). Here, using human predators, we systematically manipulated number, density and motion path predictability over a wide range in order to test the influence of each of these factors on the confusion effect. The stimulus was a novel, modified version of a standard multiple object tracking display (Pylyshyn and Storm 1988; Cavanagh and Alvarez 2005). These stimuli consist of several independently moving items, a subset of which are defined as targets whose location subjects monitor continuously and simultaneously. Our modified display tested subjects’ ability to track a single moving target object amongst many moving distractor objects.

## Methods

Stimuli were generated in MATLAB, using the Psychophysics Toolbox extensions (Brainard 1997; Pelli 1997) and displayed on a gamma-corrected monitor (Sony Trinitron) with a spatial resolution of 1024 × 768 pixels, a temporal resolution of 100 Hz, and a mean luminance of 65.5 cd/m^2^. At the experimental viewing distance of 59 cm, each pixel subtended 2 minarc.

**Fig. 1:**
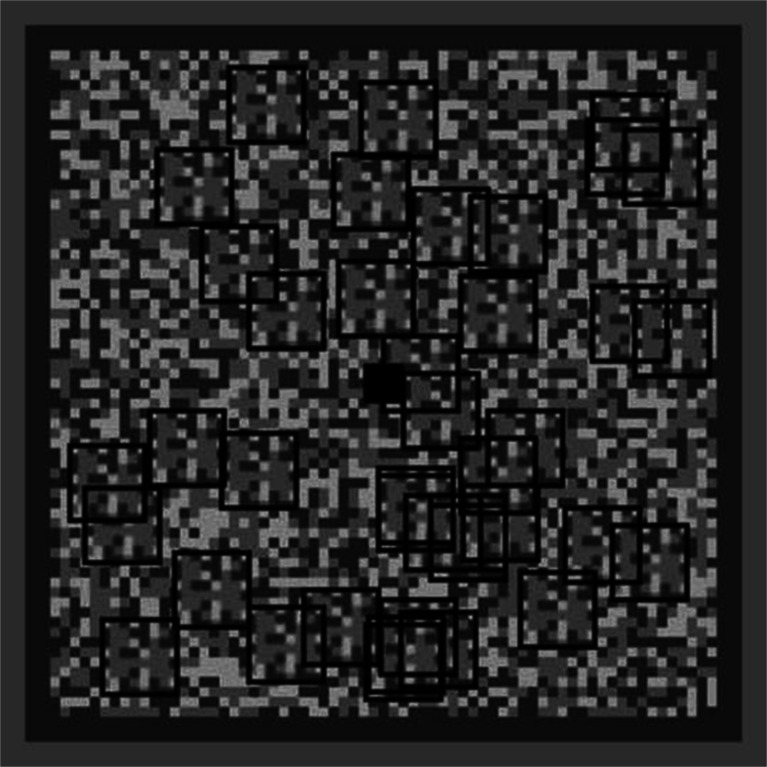
Example of the display a subject saw at the end of a trial after the objects stopped moving, prior to identifying the target by mouse click. The treatment shown here is 40 objects in a 268 × 268 pixel display, an intermediate density. The *black square* marks the initial location of the mouse-driven cursor. At the start of the trial, only the target to be tracked was framed in *black* and *white* and all objects started moving. This border remained for the first 1 s of the trial, and then was removed for the remaining 4 s

Subjects carried out a single object tracking task, inspired by the well-established multiple object tracking task (Pylyshyn and Storm 1988; Cavanagh and Alvarez 2005). The new task required subjects to monitor the location of a single moving target amongst many dynamic distractors and report its final position. On each trial, either 20, 40 or 80 objects were initially placed randomly on a square background (Fig. 1). The objects were 32 × 32 pixel (64 × 64 minarc) trinary noise squares (each pixel of an object having luminance values of 32.75, 65.5, or 98.25 cd/m^2^ with probability 1/3), and with stimulus onset, they started moving at 6.67°/s. A single target object was highlighted with a black and white border for the first 1000 ms of the trial, and then the border was removed for the next 4000 ms. The target object was plotted last, meaning that it was always completely visible in front of the distractor objects. At the end of the trial, all the objects stopped and were each surrounded by a black border. The subject’s task was to indicate, with a mouse-driven cursor, which of the objects was the initially highlighted one. The Cartesian distance between the middle of the target object and the middle of the cursor on mouse clicking was recorded on each trial.

Each object’s direction was initially determined by an independent random draw from a uniform distribution between 0° and 359.99°. Subsequently, on each frame of the stimulus presentation, direction was freshly and independently determined for each object by drawing it from a circular Gaussian distribution, centred on the previous frame’s direction. The standard deviation of the Gaussian distribution was varied in factorial combination with other treatments in seven evenly log-spaced steps: pi/2^6^, pi/2^5.5^, pi/2^5^, pi/2^4.5^, pi/2^4^, pi/2^3.5^ and pi/2^3^ radians. This range of motion path unpredictability was determined by pilot experiments. Lower values are more predictable, higher values less so.

The background was composed of the same trinary noise texture as the objects, making them perfectly background matching. This meant that the objects would have been undetectable when stationary: hence the need for the black border around each object at the end of each trial. The objects were all identical, and display size (190 × 190, 268 × 268 and 380 × 380 pixels, corresponding to 6.3 × 6.3°, 8.9 × 8.9° and 12.7 × 12.7°, respectively) was factorially combined with number of objects (20, 40 and 80 items) in order to separate the effects of both number and density on performance. The 24 naïve subjects (19 females, mean age = 19, range 18–21) were recruited from the undergraduate population at the University of Bristol, and all subjects completed all conditions.

Targeting error was recorded as the distance in pixels between the mouse click and the centre of the target. This was, perhaps unsurprisingly, bimodal (subjects either tracked the target successfully or failed), so the response variable analysed was a binary hit (click within the target) or miss. The effect of the experimental variables on the probability of hitting the target was analysed using generalized linear mixed models with binomial error and a logit link function (Knoblauch and Maloney 2012) using package lme4 (Bates et al. 2014) in R version 3.1 (R Core Team 2013). Subject was a random effect in all models; the significance of particular terms was evaluated with likelihood ratio tests comparing models with or without the factor of interest. Where confidence intervals for particular terms are presented, they were calculated using parametric bootstrapping with function bootMer in the lme4 package. Both random effects and response values were simulated conditional on the parameter estimates, using 1000 samples.

The most complex model fitted unpredictability of object path (the standard deviation of the circular Gaussian determining direction) as a quadratic polynomial, to allow for nonlinear effects, and both the number of items (three levels) and display area (three levels) as factors. The three-way interaction between these experimental variables is the effect of primary interest as it represents the interaction between the classical confusion effect (an effect of number of objects, or density, on capture success) and predictability of target motion. Subsequent models address whether main or interaction effects of item number and display area can be modelled as, simpler, linear terms or not. The other issue of major interest is whether any effect of item number and display area can be substituted by a single variable, density (items per unit area), either as a linear or nonlinear term. These models with different fixed effects were compared using the Bayesian information criterion (BIC), a low BIC being a preferable model. BIC was used rather than Akaike information criterion (AIC) because it penalises more complex models to a greater degree (Burnham and Anderson 2002, 2004), and having established what variables had effects on capture success, we sought the simplest description of their influence.

## Results

**Fig. 2:**
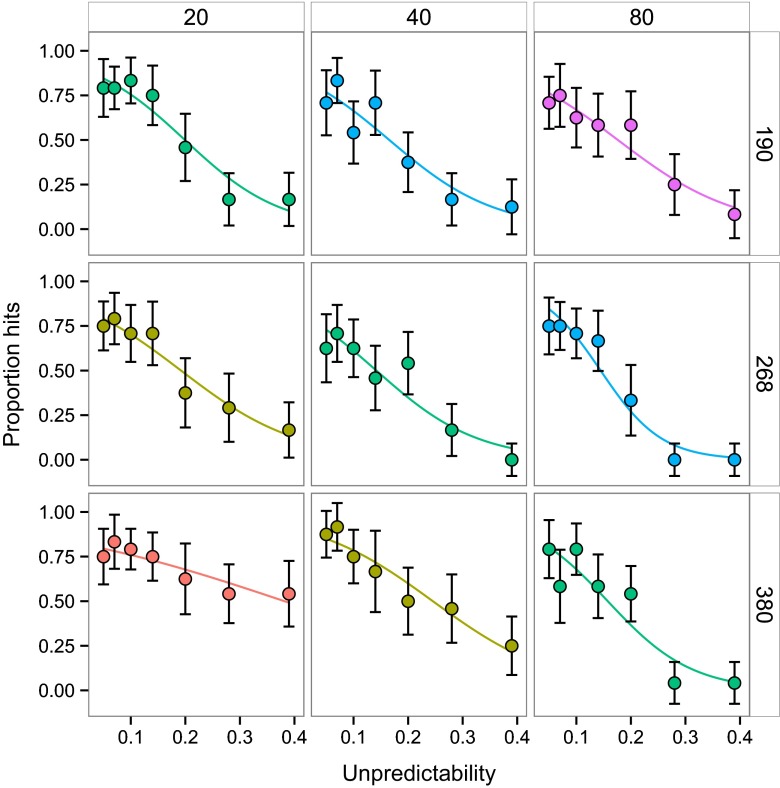
Proportion of trials where the target was successfully tracked, plotted against unpredictability of object motion. The nine *panels* represent the factorial combination of number of objects (20, 40, 80) and display area (190, 268, 380). The *error bars* are 95 % confidence intervals, with the between-subject variation removed (appropriate for a repeated-measures design). The *smooth curves* are logistic regressions. Colour codes density: *panels sharing the same colour* are the same density. The same colour code is used in Fig. 3

The unpredictability × area × items interaction was significant (likelihood ratio test: *χ* ^2^ = 27.10, *d.f.* = 8, *p* = 0.0007; Fig. 2). However, a simpler model with log(number of items), area and unpredictability as linear effects was preferable on information theoretic grounds to the full model, which treated items and area as three-level factors and unpredictability as a quadratic covariate (BIC = 1454.58 compared to 1515.25). In this simpler model, the three-way interaction was still significant (*χ* ^2^ = 10.78, *d.f.* = 1, *p* = 0.0010) and a model with it was preferable on the BIC to one without (model with only two-way interactions, BIC = 1458.04).

In a situation such as this, with a complex interaction, the typical next step would be to split the data by area and analyse each for effects of item number and unpredictability, or split by items and analyse for effects of area and unpredictability. However, we have a specific hypothesis we want to test: whether item density matters more than item number. From the fact that the area × items × unpredictability interaction is significant, we already know that item number by itself is not enough to explain the relationship between hit success and unpredictability. However, if it is density that matters, a model that fits density (either as a categorical predictor or as a continuous—but not necessarily linear—predictor) should be better than a model which allows display area and item number to vary independently.

A model with linear effects of area, log(number of items), unpredictability and their two- and three-way interactions had a BIC of 1454.58 (see above). Fitting density (number of items divided by display area) as a quadratic covariate, with unpredictability and the density × unpredictability interaction, gave a more parsimonious model (BIC = 1444.98). Fitting density as a linear predictor did not (BIC = 1476.15), and removal of the interaction also gave a poorer fit (BIC = 1456.41). In conventional hypothesis-testing terms, the quadratic density × unpredictability interaction was significant (*χ* ^2^ = 26.07, *d.f.* = 2, *p* < 0.0001).

**Fig. 3:**
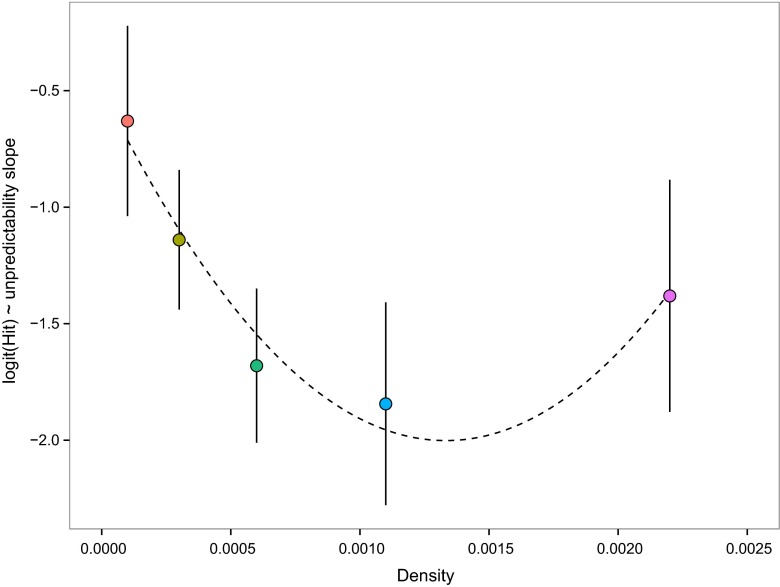
The slope (with 95 % c.i.s) of the relationship between proportion of targets successfully tracked and the unpredictability of target motion, plotted against object density. The slopes are based on logistic regressions, as in Fig. 2. The deleterious effect of unpredictability on target tracking (the magnitude of the negative slope) increases with object density, then declines at the highest density, as indicated by the plotted *quadratic curve*. The *colours of the points* correspond to those used in Fig. 2

Therefore, the best supported model is an interaction between unpredictability and a quadratic effect of density (visualised in Fig. 3). As unpredictability increased, hit rate went down, and the magnitude of that negative effect increased with distractor density, but only up to a point. At the highest density, the effect of unpredictability diminished. We note that even the shallowest relationship between hit rate and unpredictability (at the lowest density: 20 items in a display area of 380, the red points in Figs. 2 and 3) has a mean significantly different from 0 (*t*_23_ = 4.09, *p* = 0.0005). Unpredictability therefore increases targeting error for all group densities, but the magnitude of the effect increases at low-to-medium densities then reaches a ceiling at the highest density.

What of the confusion effect for targets moving completely predictably? We did not have such a treatment in our experiment, but this is estimated by the coefficients for the effect of density from the best-fitting model when the effect of unpredictability is zero (i.e. the intercept; see Fig. 2). Neither the linear coefficient of density (−0.78; 95 % c.i.s -10.43, 8.98) nor the quadratic (−7.80; 95 % c.i.s −17.94, 2.52) were significantly different from zero. No great weight should be placed on conclusions about a null effect of density based on these (broad) confidence intervals, as they estimate a parameter outside the range of the experimental values. However, analysing only the data from the lowest manipulated level of unpredictability (0.05) also shows no effect of density on hit rate (likelihood ratio test for model with quadratic effect of density vs one without: (*χ* ^2^ = 1.0689, *d.f.* = 2, *p* = 0.586).

## Discussion

Our stimuli generated no confusion effect when objects moved with predictable paths: for both estimated hit rates at zero unpredictability or at the lowest values of motion path unpredictability, object tracking performance was good and object density had no effect. At higher levels of unpredictability, a confusion effect appeared and then increased as path unpredictability increased. This was true of all nine conditions tested (Fig. 2). Ours is the first study to combine, factorially, manipulations of number of objects and the area within which the targets move. In doing so, we have shown that the higher density of larger groups more parsimoniously explains decline in targeting success than the group numbers alone. The effect, however, is nonlinear (Fig. 3): the interaction between motion predictability and the confusion effect increases with density up to a point but then reaches a ceiling, or even declines slightly, at the highest density. We suspect this is an artefact of the target always being in front of the other objects, such that at the highest density there are few gaps and so the background becomes a uniform dynamic texture rather than individuated objects. Although an artefact in the sense that real group-living animals continuously change place, in part to put other group members between themselves and the predator (Hamilton 1971), this was necessary in our experiment in order to remove the possible confounding effect of the probability of occlusion—certainly a real benefit of living in larger groups, but not the object of investigation here.

The fact that motion path unpredictability interacted with the confusion effect (a deleterious effect of increased number of objects or density on targeting) seemingly contradicts Jones et al. (2011). This difference could be attributed to stimulus differences between the two studies. The two most obvious differences are the precise form of the unpredictable motion and the appearance of the moving objects. Jones et al. (2011) manipulated turning angle in prey items which swarmed around the centre of the screen, whereas we changed predictability of movement for items which performed random walks whilst constrained by the screen boundaries. As far as appearance goes, Jones et al. (2011) used dark dots with tails on a light background, and we used background matching trinary noise squares. Background matching itself is unlikely to have had an influence, as our stimuli were immediately salient upon moving. However, Hall et al. (2013) found that target–distractor discrimination was poorer for groups of camouflage-patterned objects than for unpatterned targets. Hall et al.’s stimuli moved predictably, so how patterning and predictability of motion, both relevant factors for many group-living species, moderate or enhance the confusion effect should be investigated.

Our experimental design isolates factors affecting performance in tracking a single pre-specified target amongst many very similar distractors. This is a real world problem, not just in the interactions between predators and group living prey but, beyond biology, in human interactions (spotting your child in a playground, a pick-pocket in a crowd, a terrorist at an airport) and computer vision (notably in security and military applications). In all these domains, there is also frequently a phase prior to the process we analyse, where multiple potential targets are tracked. For example, where multiple potential prey are assessed for vulnerability or profitability, or where multiple threats need to be evaluated. This can be done serially or, to a limited degree, in parallel (Pylyshyn and Storm 1988; Cavanagh and Alvarez 2005). Multiple target tracking has therefore become an important field in computational vision as well as psychology, where it pertains to issues such as the degree to which attention can be divided. The extent to which unpredictable motion and prey density during the target assessment phase contribute to the classical confusion effects therefore deserves further scrutiny. In this paper, we have outlined a paradigm that is well suited to this task, with relevance to biology and the human visual sciences.
